# Evaluation of Rice Straw, Corncob, and Soybean Straw as Substrates for the Cultivation of *Lepista sordida*

**DOI:** 10.3390/life14010101

**Published:** 2024-01-08

**Authors:** Chunge Sheng, Yanfeng Wang, Chunlei Pan, Lei Shi, Yuanhang Wang, Yinpeng Ma, Jinhe Wang, Jing Zhao, Peng Zhang, Zitong Liu, Haiyang Yu, Fei Wang, Xuemei Dong, Shuihua Yan

**Affiliations:** 1 Mudanjiang Branch, Heilongjiang Academy of Agricultural Sciences, Mudanjiang 157000, China; shengchunge@haas.cn (C.S.);; 2 College of Resources and Environmental Sciences, Northeast Agricultural University, Harbin 150030, China; 3 Institute of Microbiology, Heilongjiang Academy of Sciences, Harbin 150010, China; 4 College of Pharmacy, Mudanjiang Medical University, Mudanjiang 157011, China

**Keywords:** mushroom cultivation, *Lepista sordida*, grain crop residues, substrates

## Abstract

*Lepista sordida* is a type of high-quality rare edible and medicinal mushroom, and its research boom is just beginning. More than 80 million tons of grain crop residues are produced each year in Heilongjiang Province. To realize the exploration and utilization of wild *L. sordida* mushrooms and also provide a theoretical support for the high-value utilization of these resources in Heilongjiang Province, we evaluated the cultivation of *L. sordida* mushrooms using rice straw, corncob, and soybean straw as substrates. *L. sordida* grew on all three substrates, and the biological efficiency and yield of the mushrooms grown on soybean straw and corncob were 32.33 ± 1.78% and 4.20 ± 0.23 kg m^−2^, and 30.15 ± 0.93% and 3.92 ± 0.12 kg m^−2^, respectively, which increased by 9.38% and 2.08% compared with that on the rice straw substrate with 3.84 ± 0.12 kg m^−2^ and 29.56 ± 0.89%. The time it took for the mycelia to colonize and initiate primordia on the soybean straw substrate was 22.33 ± 0.58 d and 19.67 ± 0.58 d, respectively, which was delayed by 2 d and 3 d compared with that on the rice straw substrate with 20.67 ± 2.08 d and 16.33 ± 0.58 d, respectively. The fruiting bodies grown on corncob and soybean straw substrates were relatively larger than those on the rice straw substrate. The highest amount of crude protein was 57.38 ± 0.08 g 100 g^−1^, and the lowest amount of crude polysaccharide was 6.03 ± 0.01 g 100 g^−1^. They were observed on mushrooms collected from the corncob substrate. The contents of the heavy metal mercury, lead, arsenic, and cadmium in the fruiting bodies grown on each substrate were within the national safety range.

## 1. Introduction

*Lepista sordida* is an edible mushroom that mostly grows on the ground in gardens, lawns, or parks [1] and belongs to the Tricholomataceae [2]. This fungus is widely distributed in nature. Currently, *L. sordida* has been shown to be broadly dispersed in Europe, Asia, Africa, Australia, and North America [3]. *L. sordida* is primarily distributed in the northeast, central, southern, and northern regions of China [4]. The morphological features of *L. sordida* are very similar to those of the well-known violet *L. nuda* [5], particularly when the mushrooms are mature. Thus, it is difficult to tell them apart [6]. As a result, this similarity has the potential to lead to their misidentification, which is common both between and within the *Lepista* species [7].

This mushroom provides various major nutrients [8], such as vitamins, proteins, amino acids, dietary fiber, carbohydrates, and less fat. These beneficial compounds are of great interest to the food industries and pharmaceutical sectors. This cultivation of this species provides high-quality additional vegetables for consumers and enriches their diets, which can directly benefit fitness and health. It has great potential for exploitation as a pivotal nutritional supplement [9] in diets and thus has turned out to be exceptional and difficult to replace. As a functional edible fungus, *L. sordida* is globally appreciated. However, the properties of this fungus remain to be thoroughly explored.

China was the first country to domesticate the *L. sordida* mushroom as early as 1981 [10] and successfully obtained the fruiting body of this mushroom using a manure–grass substrate. Lu et al. [11] in 1994 and Xiao et al. [12] in 1995 elaborated on the environmental conditions and carbon (C) and nitrogen (N) nutrition requirements of the species. Since the beginning of the 21st century, research on the techniques of cultivating *L. sordida* has become more active in China. Research by Tian et al. [13] showed that the best cultivation mode of *L. sordida* is the method of clinker cultivation; the optimal substrate was a mixture of 56% rice (*Oryza sativa* L.) straw (wheat (*Triticum aestivum* L.) straw) and 40% dry chicken manure, and the biological efficiency (BE) was 19.9% on this medium. The closest to the level of commercial production for this species was research by Li et al. [14] from Taiwan Province in 2014. In their research, they confirmed a 43% BE of this mushroom cultivated on *Agaricus bisporus* compost. Lun et al. [15] explained 36.8% and 28.4% of the BE using corn stalk and corncob substrates, respectively. Zhou et al. [16] used mixed materials (40% fresh cow dung + 20% turf powder + 20% rice bran + 18% sawdust) as the substrate to cultivate this mushroom and proposed a yield of 21.1 g bag^−1^. According to information available in English, Tongbai et al. [3] from Thailand were the first people to report the successful cultivation of *L. sordida* in 2017 with a yield of 287.5 g kg^−1^. The Chinese researchers Xu et al. [17] proposed a corncob-based substrate and explained 41.22% of the BE of this mushroom, while Sheng et al. [18] confirmed a yield of 4.51 ± 0.65 kg m^−2^ and a BE of 34.69%, as well as contents of 25.64 ± 0.38 g 100 g^−^^1^ of crude polysaccharides in the fruiting bodies collected from the spent substrates of *Auricularia heimuer* (SSA) in 2023. To date, the cultivation of *L. sordida* mushrooms is still in the exploratory stage, which greatly limits the promotion of *L. sordida* mushrooms. There are still many problems, such as low yield and unstable traits, that need to be solved in the cultivation of *L. sordida* mushrooms. *L. sordida* has not yet been cultivated commercially [14], and consumers still cannot find *L. sordida* products in local and foreign markets.

Heilongjiang Province is China’s largest producer of grain and exporter of commodity crops and is located in Northeast China. Corn, rice, and soybean (*Glycine max* L.) are the three largest crops in Heilongjiang Province [19]. Data from the National Bureau of Statistics of China (http://www.stats.gov.cn/) indicate that more than 95 million tons of grain crop residues were produced with 81 million tons that could be collected in Heilongjiang Province in 2021. These residues are a non-competitive resource. Its high-value, resource-based utilization has been a global hot issue. In China, grain crop residues are known as “The other half of agriculture”. If these residues are well utilized, they will be a treasure; if not, they will be harmful to humans and the environment. Realizing the harmless and sustainable utilization of grain crop residues is a very important and urgent problem that needs to be solved. These residues are primarily composed of cellulose, hemicellulose, and lignin [20], which provide nutrients for the growth and cultivation of mushrooms; thus, grain crop residues are a potential substrate for mushroom cultivation [21,22]. Utilizing such residues to cultivate edible fungi will enhance the utilization of waste and decrease environmental pollution.

This study first evaluated the effects of different types of substrates based on grain crop residues on the mycelial growth, primordial initiation time, fruiting body morphology, yield, BE, nutrient composition, and heavy metals of *L. sordida*, which not only provides a new substrate material for highly efficient and high-quality cultivation but also provides a good and feasible way to utilize agricultural waste.

## 2. Materials and Methods

### 2.1. Lepista Sordida Strain

**Figure 1:**
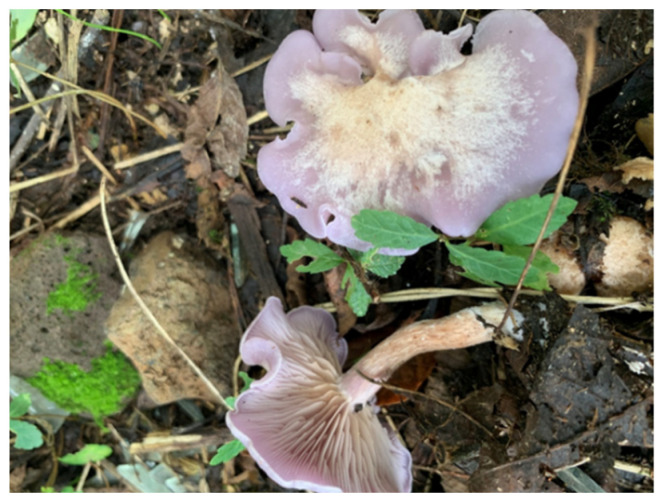
Wild fruiting body of *Lepista sordida*.

The *L. sordida* (ZD4) strain used in this research was preserved in the Mudanjiang Branch of Heilongjiang Academy of Agricultural Sciences (Mudanjiang, China). This strain was obtained from a wild one (Figure 1), whose fruiting bodies were collected from the National Forest Park (Sandaoguan, Mudanjiang), Heilongjiang Province, Northeast China, in August 2018. Pure cultures were incubated on potato dextrose agar (PDA) media at 25 °C.

### 2.2. Molecular Biological Verification of the Wild Strain

The primers ITS1 and ITS4 (ITS1, 5′-TCCGTAGGTGAACCTGCGG-3′; ITS4, 5′-TCCTCCGCTTATTATTGATATGC-3′) that were used for amplification were synthesized by Sangon Biotech Co., Ltd. (Shanghai, China). A volume of 25 µL of the PCR mixture was used for this experiment. It contained 1.0 μL of each type of primer, 1.0 μL of a DNA template, 2 µL of dNTPs, 0.5 µL of Taq enzyme (Tiangen Biotech, Beijing Co., Ltd., Beijing, China), and 2.5 µL of a 10× Tap buffer. The PCR was performed with a touchdown program that utilized the following parameters: 4 min at 95 °C; 30 s at 94 °C; 30 s at 57 °C; 90 s at 72 °C; a cycle of 30 times; and 10 min at 72 °C. The DNA was sequenced by Sangon Biotech Co., Ltd. (Shanghai, China). The results of sequencing were analyzed using BLAST to determine their status of classification. The newly generated sequences in this study were submitted to GenBank to obtain the gene login number. Moreover, a phylogenetic tree was constructed to analyze the phylogenetic relationships of these genes. It utilized a maximum likelihood (ML) analysis in MEGA X.

### 2.3. Substrate Preparation

The rice straw, corncob, and soybean straw that were used in this study were obtained from the Mudanjiang Branch of Heilongjiang Academy of Agricultural Sciences, and the other materials were purchased from a local establishment. The crop branches were all cut into 5–10 cm pieces using a crusher (9F40-28, Xingyang, China). As shown in Table 1, T1, T2, and T3 were added with 56% rice straw, corncob, and soybean straw, respectively. All the treatments contained 40% of cow dung, 2% of lime, 1% of gypsum, and 1% of calcium superphosphate, which is a slight modification of Tian [13]. These ingredients were prepared utilizing the percentages described in the formula. The corn and soybean straws and the corncobs were pre-wetted. The material was evenly mixed and then heaped up for fermentation as described in our previous study [18]. The water contents in the final mixtures were modified to range from 55% to 65% (*w*/*w*).

### 2.4. Proximate Components of the Main Substrates

The contents of total nitrogen (TN) and total carbon (TC) of the raw materials were analyzed through the Kjeldahl methods and loss of ignition, respectively. The C/N is the ratio of the content of TC in the samples to that of the TN. The contents of ash-free acid detergent fiber (ADF), ash-free neutral detergent fiber (NDF) [23], and lignin [24] were determined as previously described.

### 2.5. Cultivation Methods and Investigation of Agronomic Traits

Suitable environmental conditions, such as temperature and humidity among others, and the sampling and recording methods required in each period of cultivation were conducted as described by Sheng et al. [18]. In short, the greenhouse was maintained at 22–26 °C and 80–90% relative humidity during the times of the initiation of primordia and the growth of fruiting bodies. The fermentative ridge cultivation model was used to cultivate *L. sordida* in the greenhouse. The prepared substrates were placed in each treatment with an area of cultivation of 15 m^2^ at a density of 13 kg m^−2^ (dry weight). Three replicates of the 15 m^2^ ridges were established in each treatment and randomly distributed in the greenhouse, and the morphology of the fruiting body, including the diameter and thickness of the cap and the diameter and length of stipe, was measured (cm) from 30 samples randomly selected from the first flush of fruiting bodies. The yield of mushrooms was calculated using the total fresh weight of the three flushes in each treatment of the mushrooms.

### 2.6. Chemical Analysis

Heilongjiang Huace Testing International Corporation (Harbin, China) conducted the chemical analyses, which involved determining the crude polysaccharide (NY/T 1676–2023) [25], crude fat (GB 5009.6) [26], crude fiber (GB/T 5009.10) [27], crude protein (GB 5009.5) [28], and ash (GB 5009.4) [29] as described in the National Food Safety Standards. The content of crude protein (N × 4.38) was measured through the macro Kjeldahl method [30]. The heavy metals lead (Pb), arsenic (As), mercury (Hg), and cadmium (Cd) were analyzed using the Chinese standard methods for Pb (GB 5009.12) [31], As (GB 5009.11) [32], Hg (GB 5009.17) [33], and Cd (GB 5009.15) [34] issued by the Standardization Administration of China.

### 2.7. Statistical Analysis

Microsoft Excel 2019 (Redmond, WA, USA) was used to process the raw data. The statistical analyses were conducted using SPSS 26.0 (IBM, Inc., Armonk, NY, USA). The data obtained were expressed as the mean ± SD (standard deviation). LSD multiple range tests at a 95% confidence level (*p* < 0.05) were used to assess the differences among the means of each treatment. GraphPad Prism 8 (San Diego, CA, USA) was used to plot the figures and conduct heat mapping.

## 3. Results

The identification of the strain and its phylogeny, components of the main substrates, mycelial growth and development, fruiting body morphology, yield, BE, nutrient composition, and heavy metals of *L. sordida* was as follows: 

### 3.1. Strain Identification and Phylogeny

**Figure 2:**
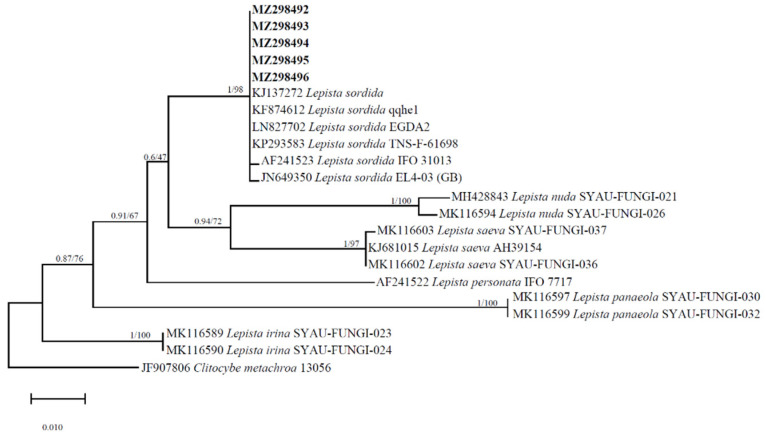
The molecular phylogenetic tree of the ITS regions of *Lepista sordida* as evinced by maximum likelihood and Bayesian analyses. The support values are given at the branches. The bar represents the number of expected substitutions per position.

The ITS nucleotide sequences in GenBank revealed that the most similar sequences were from *L. sordida* JX434648.1 from China at 99.85% similarity and 100% query cover. A 657 bp ITS sequence of the strain was deposited in GenBank with accession number MZ298494 (https://www.ncbi.nlm.nih.gov/nuccore/MZ298494.1 Accessed on 20 September 2023). The molecular phylogenetic trees show that the new ZD4 collection and the other collections of *L. sordida* grouped together with a high statistical support value (BPP = 1, ML = 98) (Figure 2). All the species in this clade belong to that of *L. sordida*. Based on the phenotypic characteristics, BLAST comparison, and phylogenetic analysis results, we clearly identified this strain as *L. sordida.*

### 3.2. Components of the Main Substrates

Three replicates of each substrate were conducted, and the results are shown as the mean ± SD. The composition and contents in the main raw materials for *L. sordida* cultivation are listed in Table 2. The cellulose contents of several raw materials ranged from 33.13 ± 1.84 to 38.53 ± 1.38. Corncob had the highest cellulose content, and its hemicellulose content ranged from 14.21 ± 0.56 to 26.27 ± 0.92. Soybean straw had the lowest hemicellulose content, and rice straw had the highest hemicellulose content. The lignin content of the soybean straw was higher with the value of 27.65 ± 0.89, and that of corncob was lower at 5.94 ± 0.59. The content of TC in the rice straw was higher at 41.50 ± 2.00, and the content of TN in the soybean straw was higher at 1.24 ± 0.05. The C/N ratio of rice straw, corncob, and soybean stalk was 65.87, 88.00, and 32.46, respectively.

### 3.3. Mycelial Growth and Development

**Figure 3:**
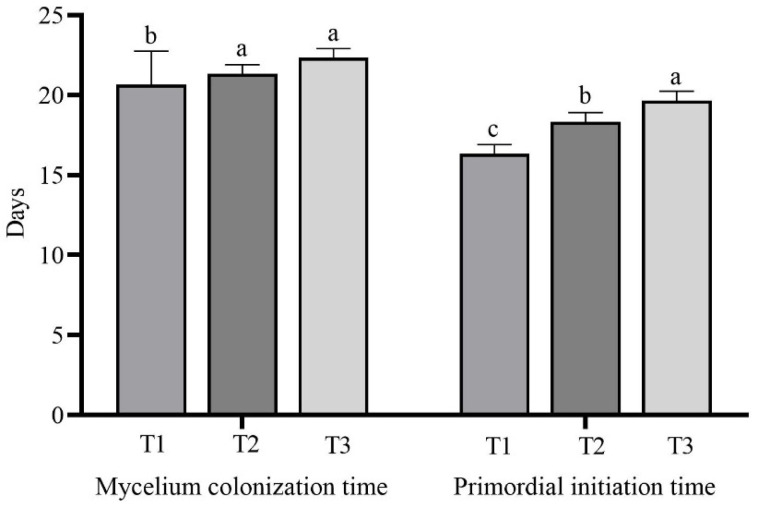
Mycelial growth and development in each treatment. The means ± SD are shown. Different case letters above each bar represent significant differences (α = 0.05, ANOVA, LSD test). T1: 56% rice straw, 40% cow dung, 2% lime, 1% gypsum, and 1% calcium superphosphate; T2: 56% corncob, 40% cow dung, 2% lime, 1% gypsum, and 1% calcium superphosphate; T3: 56% soybean straw, 40% cow dung, 2% lime, 1% gypsum, and 1% calcium superphosphate. ANOVA, one-way analysis of variance; LSD, least significant difference.

The number of days for the mycelia of *L. sordida* to colonize different treatment substrates and initiate primordia is shown in Figure 3 and Table S1. The mycelial colonization time of the T1 treatment with 56% rice straw, the T2 treatment with 56% corncob, and the T3 treatment with 56% soybean straw was 20.67 ± 2.08 d, 21.33 ± 0.58 d, and 22.33 ± 0.58 d, respectively. The mycelia in T1 treatment colonized the substrate substantially more quickly than the T2 and T3 treatments, which did not differ significantly from each other. The mycelia took the longest time to colonize the substrate in the T3 treatment that contained 56% soybean straw. There was a 6 d delay compared with the T1 treatment with 56% rice straw.

The primordia required 16.33 ± 0.58 d, 18.33 ± 0.58 d, and 19.67 ± 0.58 d for initiation on the T1 treatment with 56% rice straw, T2 treatment with 56% corncob, and T3 treatment with 56% soybean straw, respectively. There was a significant delay in the days required for the primordial initiation among each treatment group with different substrates, and the longest was the T3 treatment with 56% soybean straw. In this treatment, the initiation was significantly delayed by 3 d compared with the T1 treatment with 56% rice straw.

### 3.4. Fruiting of Lepista sordida Grown on Different Substrates

**Figure 4:**
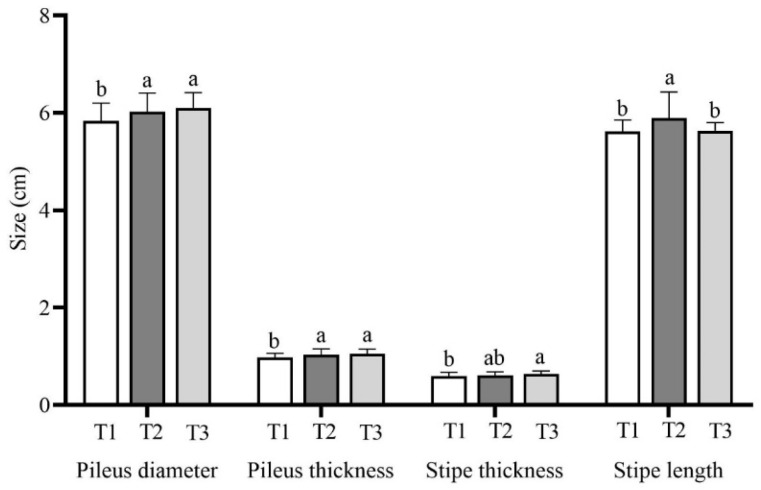
Fruiting body morphology of *Lepista sordida* mushrooms grown on different substrates. The means ± SD are shown. Different case letters above each bar represent significant differences (α = 0.05, ANOVA, LSD test). T1: 56% rice straw, 40% cow dung, 2% lime, 1% gypsum, and 1% calcium superphosphate; T2: 56% corncob, 40% cow dung, 2% lime, 1% gypsum, and 1% calcium superphosphate; T3: 56% soybean straw, 40% cow dung, 2% lime, 1% gypsum, and 1% calcium superphosphate. ANOVA, one-way analysis of variance; LSD, least significant difference.

The fruiting body morphology data, including the cap diameter, cap thickness, stipe diameter, and stipe length, are listed in Figure 4 and Table S1. The T1 treatment with 56% rice straw had a significantly smaller cap diameter (5.83 ± 0.37 cm), which was significantly lower than those of the T2 and T3 groups with 6.02 ± 0.39 cm and 6.10 ± 0.31 cm, respectively. The cap thickness of T1 was 0.97 ± 0.09 cm, which was significantly smaller than that of T2 at 1.04 ± 0.12 cm and T3 at 1.05 ± 0.09 cm. The stipe was 0.63 ± 0.06 cm, 0.61 ± 0.07 cm, and 0.59 ± 0.08 cm thick, respectively. The stipe was significantly thicker in the T3 treatment than in the T1 treatment, and there was no significant difference between the T2 treatment and the other treatments. The stipe in T2 treatment was 5.89 ± 0.54 cm long, which was significantly longer than that in the T1 treatment with 5.62 ± 0.23 cm and the T3 treatment with 5.63 ± 0.17 cm. 

### 3.5. Yield and Biological Efficiency 

**Figure 5:**
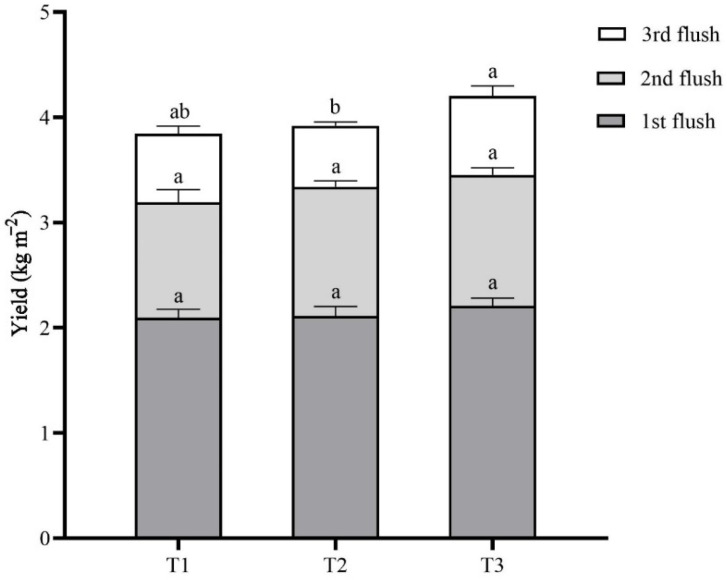
Accumulative bar diagram of the yield distribution. Different case letters above each bar represent significant differences (α = 0.05, ANOVA, LSD test). T1: 56% rice straw, 40% cow dung, 2% lime, 1% gypsum, and 1% calcium superphosphate; T2: 56% corncob, 40% cow dung, 2% lime, 1% gypsum, and 1% calcium superphosphate; T3: 56% soybean straw, 40% cow dung, 2% lime, 1% gypsum, and 1% calcium superphosphate. ANOVA, one-way analysis of variance; LSD, least significant difference.

Three flushes of *L. sordida* mushrooms were harvested, and the total fresh weight was determined. As shown in Figure 5 and Table 3, the total yield of the *L. sordida* mushrooms collected from the T1, T2, and T3 groups was 3.84 ± 0.12 kg m^−2^, 3.92 ± 0.12 kg m^−2^, and 4.20 ± 0.23 kg m^−2^, respectively. The highest total yield was observed in the T3 treatment, and it was significantly higher than those of the T1 treatments. 

The yield of the first flush of *L. sordida* mushrooms in each treatment ranged from 2.10 ± 0.08 kg m^−2^ to 2.21 ± 0.08 kg m^−2^. There was no difference in the first flush of mushrooms among the T1, T2, and T3 groups. The yield of the second flush of *L. sordida* mushrooms ranged from 1.10 ± 0.12 kg m^−2^ to 1.25 ± 0.07 kg m^−2^. In addition, there was no difference among each group. There was a significant effect from the different substrates on the yield of the third flush of *L. sordida* mushrooms. The T3 treatment gave a significantly higher yield of 0.75 ± 0.10 kg m^−2^ than that of the T2 treatment of 0.58 ± 0.04 kg m^−2^. The yield of T1 treatment was 0.65 ± 0.07 kg m^−2^, which has no significant difference from the other two treatments.

The BE of mushrooms cultivated on the T1, T2, and T3 treatments was 29.56 ± 0.89%, 30.15 ± 0.93%, and 32.33 ± 1.78%, respectively. The highest BE was observed in the T3 group, and it increased significantly by 9.37% compared with that of the T1 group.

### 3.6. Nutritional Components of the Lepista sordida Mushrooms

**Figure 6:**
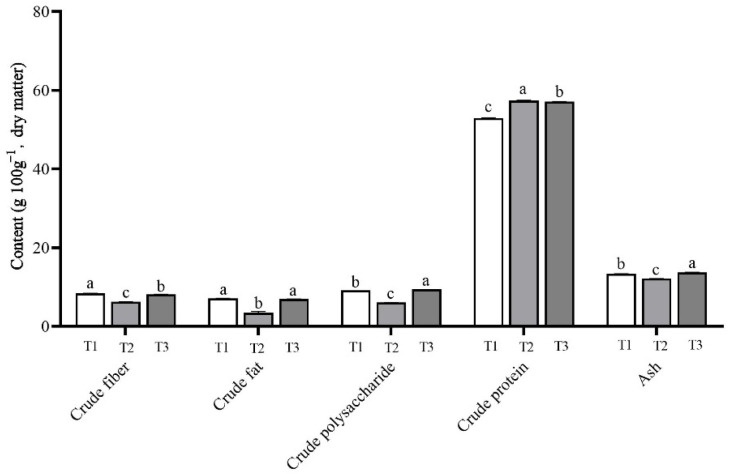
Nutritional components of *Lepista sordida* mushrooms on different substrates (g 100 g^−1^ of dry matter). Different case letters above each bar represent significant differences (α = 0.05, ANOVA, LSD test). T1: 56% rice straw, 40% cow dung, 2% lime, 1% gypsum, and 1% calcium superphosphate; T2: 56% corncob, 40% cow dung, 2% lime, 1% gypsum, and 1% calcium superphosphate; T3: 56% soybean straw, 40% cow dung, 2% lime, 1% gypsum, and 1% calcium superphosphate. ANOVA, one-way analysis of variance; LSD, least significant difference.

As shown in Table S2 and Figure 6, there was a significant difference in the content of nutrients in the *L. sordida* fruiting bodies grown on different substrates. In this study, the mushrooms that were grown on the T1 treatment with 56% rice straw had the highest crude fiber and crude fat contents, the lowest crude protein content, and the moderate crude polysaccharide and ash content. Moreover, the lowest contents of crude fiber, crude polysaccharide, crude fat, and ash and the highest content of crude protein were observed on the samples grown on the T2 treatment with 56% corncob substrate. The T3 treatment with 56% soybean straw produced the highest contents of crude polysaccharide and ash and moderate contents of crude fiber, crude fat, and crude protein.

The samples grown on the T3 treatment had the highest content of crude polysaccharide at 9.39 ± 0.01 g 100 g^−1^. This was a significant increase of 55.72% compared with the 6.03 ± 0.01 g 100 g^−1^ of the T2 group. The content of crude protein of 57.38 ± 0.08 g 100 g^−1^ in the fruiting bodies harvested from the T2 group increased significantly by 8.41% compared with that of 52.93 ± 0.07 g 100 g^−1^ in the T1 group. 

### 3.7. The Heavy Metal Composition of the Mushrooms

The contents of heavy metals were also determined to analyze the effect of different substrates on the fruiting bodies. As shown in Table 4, there were values of As at 0.37 ± 0.01 mg kg^−1^, Pb at 0.30 ± 0.02 mg kg^−1^, Cd at 0.44 ± 0.03 mg kg^−1^, and Hg at 0.039 ± 0.001 mg kg^−1^ in the T1 samples. The T2 samples had values of As at 0.53 ± 0.01 mg kg^−1^, Pb at 0.55 ± 0.01 mg kg^−1^, Cd at 0.46 ± 0.01 mg kg^−1^, and Hg at 0.143 ± 0.006 mg kg^−1^, while those of the T3 samples were As at 0.47 ± 0.01 mg kg^−1^, Pb at 0.43 ± 0.02 mg kg^−1^, Cd at 0.45 ± 0.01 mg kg^−1^, and Hg at 0.140 ± 0 mg kg^−1^.

This is an interesting set of objective data. Except for Cd, there were substantial effects on the different substrates that were used for cultivation based on the composition of heavy metals, including As, Pb, and Hg. Additionally, we found that the samples cultivated on the T1 treatment with 56% rice straw provided the lowest contents of As, Pb, Cd, and Hg. Otherwise, the highest contents of the heavy metals As, Pb, Cd, and Hg were recorded in the samples cultivated on the T2 treatment with 56% corncob, and the T3 treatment with 56% soybean straw provided moderate contents of As, Pb, Cd, and Hg. 

## 4. Discussion

There were significant effects of the different cultivation substrates on the yields, biological efficiency (BE), and morphology of the fruiting bodies. We found that the fruiting bodies grown on corncob and soybean straw substrates had higher yields and BE and larger fruiting bodies than those of the mushrooms that grew on the rice straw substrates. In practical mushroom production, the yield and BE are the most important and fundamental primary traits, and the nutritional composition of the cultivation substrate and the environmental conditions directly influence these parameters [35,36]. In this study, *L. sordida* was successfully cultivated on rice straw, corncob, and soybean straw substrates. Although the yield and BE of *L. sordida* on the soybean straw substrate were higher compared with the rice straw substrate, the yield was still lower compared to our previous research in which the highest yield reached as high as 4.51 ± 0.65 kg m^−2^ [18]. This proves that the cultivation of the mushroom in corncob, soybean straw, and composite materials still merits further study to achieve higher yields and BE, and shorten the distance with the commercial production of *L. sordida.*

The first, second, and third flushes of the *L. sordida* mushrooms comprised 50%, 30%, and 20% of the total yield, respectively, and there were no significant correlations between the mycelial growth and development and total yield. These results are similar to our previous study on this species cultivated on an SSA substrate [18].

**Figure 7:**
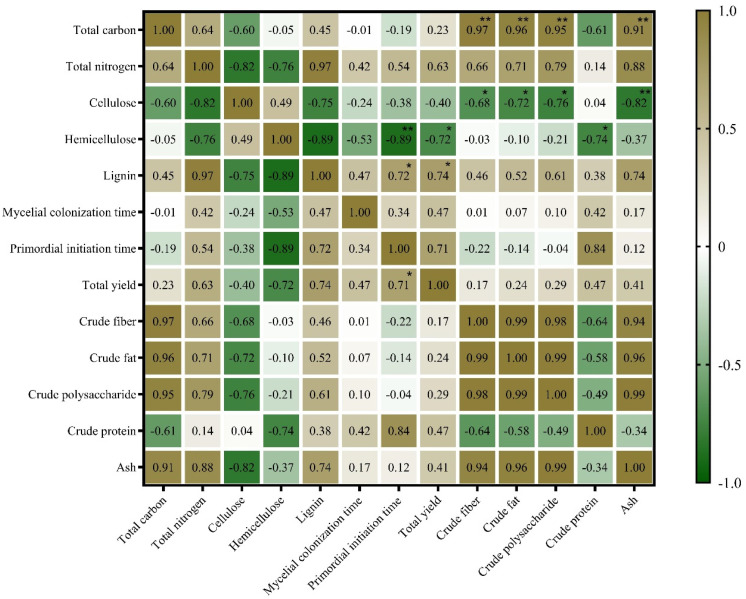
A heatmap of the correlation of cultivation substrates and characteristics and nutrient components of the *Lepista sordida* strain. The green and brown in the heat map indicate the degree of association. Brown, positive correlation. Green, negative correlation. * *p* < 0.05. ** *p* < 0.01.

The edible fungi obtain C, N, vitamins, and mineral elements from the compost, and the C content in the compost primarily comes from lignin and cellulose of the substrate. Owing to the difference in the contents of lignin, cellulose, and hemicellulose in different media, different C and N sources will affect the ability of edible fungi to decompose the substrate [37,38]. The primary ingredients in these substrates are the key factors that affect the production and accumulation of nutrients of the fruiting bodies of the edible fungi [39]. In this study, a correlation analysis (Figure 7) was used to show the effect of the nutritional composition in the substrate on mycelial growth, fruiting body yield, and nutritional quality of the *L. sordida* mushrooms. The primordial initiation time and total yield of this species negatively correlated with the content of hemicellulose in the substrate. However, they positively correlated with the content of lignin in the substrate. These results are similar to those of Rizki et al. [40], who found that a higher content of lignin in the substrate inhibited the initiation of primordia but promoted high yields.

An *L. sordida* polysaccharide has been shown to be effective against cancer [41] and possess immune-regulatory [42], anti-aging [43], antioxidative, and hepatoprotective [44] properties in vitro and in vivo from both submerged cultures and fruiting bodies. In this study, the contents of crude polysaccharide varied from 6.03 ± 0.01 g 100 g^−1^ to 9.39 ± 0.01 g 100 g^−1^, although these values exceeded those of *Pleurotus citrinopileatus* [45] cultivated on common reed substrates and *Pleurotus eryngii* [46] cultivated on the Korshinsk pea shrub (*Caragana korshinskii* Kom.). However, these values of crude polysaccharides were much lower than that of the *L. sordida* cultivated on the spent substrate of *Auricularia heimuer* in which the content of crude polysaccharide in the *L. sordida* fruiting bodies was 25.64 ± 0.38 g 100 g^−1^ [18].

In addition, the contents of crude protein varied from 52.93 ± 0.07 g 100 g^−1^ to 57.38 ± 0.08 g 100 g^−1^, which exceeded those of *Oudemansiella raphanipes* [47], *Pholiota microspore* [48], and *Pleurotus* spp. mushrooms [30]. These values also exceeded those of Lu et al. [49] and our previous study [18]. This is also the highest reported on the crude content of this mushroom. Our results are similar to those of Reis et al., who showed that the nutrients of the mushroom are affected by the species [50] and substrate [51,52,53,54]. Our current results also prove that this mushroom has a high nutritional value and meets the needs of modern consumers for nutrition and health. 

As shown in Figure 7, a heatmap showed that the contents of crude fiber, crude polysaccharide, crude fat, and ash of the *L. sordida* fruiting bodies positively correlated with the content of TC in the substrate and negatively correlated with the content of cellulose in the substrate. However, the contents of crude protein and hemicellulose in the matrix were significantly negatively correlated. This result is inconsistent with those of Zhou et al. [20], who reported that the contents of C and N in the medium had less effect on the contents of ash, polysaccharide, and fat of the mushrooms. We hypothesized that the reason may be that the nutrient composition of the fruiting body is not only related to the cultivation substrates but also to the strain genotypes and cultivation conditions among other factors, which was also reported by Xu et al. [55].

Data have shown that the mushrooms have a specific mechanism that enables them to effectively absorb heavy metals from the environment [56]. The concentration of heavy metals in the mushrooms was found to considerably exceed those in agricultural crops, fruits, and vegetables [30]. Liu et al. [57] indicated that As, Hg, and Cd pose greater risks to human health than the other heavy metals. The contents of As, Pb, Cd, and Hg in this study varied from 0.37 ± 0.01 mg kg^−1^ to 0.53 ± 0.01 mg kg^−1^, 0.30 ± 0.02 mg kg^−1^ to 0.55 ± 0.01 mg kg^−1^, 0.44 ± 0.03 mg kg^−1^ to 0.46 ± 0.01 mg kg^−1^, and 0.039 ± 0.001 mg kg^−1^ to 0.143 ± 0.006 mg kg^−1^, respectively. These results are far lower than those of Luo et al., who found values of Cd at 0.58 mg kg^−1^, Pb at 0.88 mg kg^−1^, As at 1.0 mg kg^−1^, and Hg at 0.40 mg kg^−1^ [5]. These results are also lower than those of Li et al. [6] with Pb at 0.64 mg kg^−1^ and As at 0.54 mg kg^−1^, except for Cd with 0.20 mg kg^−1^. The regulations on the maximum levels of contaminants in foods as described by the State Standard of the People’s Republic China (GB2762-2022) [58] indicated that the contents of Hg, Pb, Cd, and As observed in this study were significantly lower than the limits described as safe. This suggests that using all three types of grain crop residues as substrates to cultivate mushrooms is safe for both humans and the environment.

## 5. Conclusions

We assessed the effects of rice straw, corncob, and soybean straw substrates on the BE, growth period, nutritional value, yield, and heavy metal contents of the edible mushroom *L. sordida*. The *L. sordida* mushroom varied in its characteristics of growth when different substrates were used. Our results indicated that the contents of Pb, Hg, Cd, and As observed in this study were significantly lower than the limits of safety. The soybean straw and corncob substrate improved the total yield and BE of the mushrooms, and the fruiting bodies collected from the corncob substrate have the highest accumulation of protein. The grain crop residue substrate shows great potential for use in cultivating *L. sordida* mushrooms. However, the single and mixed substrates that were used to improve the yield and quality of *L. sordida* mushrooms merit further study.

## Figures and Tables

**Table 1:** Composition of the substrate for *Lepista sordida* cultivation (DM).

Material	Treatment Group		
	T1	T2	T3
Rice straw	56	0	0
Corncob	0	56	0
Soybean straw	0	0	56
Cow dung	40	40	40
Lime	2	2	2
Gypsum	1	1	1
Calcium superphosphate	1	1	1

Note: DM, dry matter; T1: 56% rice straw, 40% cow dung, 2% lime, 1% gypsum, and 1% calcium superphosphate; T2: 56% corncob, 40% cow dung, 2% lime, 1% gypsum, and 1% calcium superphosphate; T3: 56% soybean straw, 40% cow dung, 2% lime, 1% gypsum, and 1% calcium superphosphate. T, treatment.

**Table 2:** Analysis of the chemical composition used for *Lepista sordida* cultivation (DM).

Material	Cellulose (%)	Hemicellulose (%)	Lignin (%)	C (%)	N (%)	C/N
Rice straw	35.94 ± 1.58	26.27 ± 0.92	7.57 ± 0.47	41.50 ± 2.00	0.63 ± 0.03	65.87:1
Corncob	38.53± 1.38	21.73 ± 1.10	5.94 ± 0.59	30.80 ± 1.43	0.35 ± 0.03	88.00:1
Soybean stalks	33.13 ± 1.84	14.21 ± 0.56	27.65 ± 0.89	40.25 ± 0.95	1.24 ± 0.05	32.46:1

Note: DM, dry matter; C, carbon; N, nitrogen.

**Table 3:** Fresh weight and biological efficiency (BE) of the *Lepista sordida* mushrooms grown on different substrates (means ± SD).

Treatment Group	Fresh Weight of the Mushrooms by Flushes (kg m^−2^)				BE (%)
	First Flush	Second Flush	Third Flush	Total Fresh Weight (kg m^−2^)	
T1	2.10 ± 0.08 a	1.10 ± 0.12 a	0.65 ± 0.07 ab	3.84 ± 0.12 b	29.56 ± 0.89 b
T2	2.11 ± 0.09 a	1.23 ± 0.06 a	0.58 ± 0.04 b	3.92 ± 0.12 ab	30.15 ± 0.93 ab
T3	2.21 ± 0.08 a	1.25 ± 0.07 a	0.75 ± 0.10 a	4.20 ± 0.23 a	32.33 ± 1.78 a

Note: Different case letters represent significant differences (*p* < 0.05). T1: 56% rice straw, 40% cow dung, 2% lime, 1% gypsum, and 1% calcium superphosphate; T2: 56% corncob, 40% cow dung, 2% lime, 1% gypsum, and 1% calcium superphosphate; T3: 56% soybean straw, 40% cow dung, 2% lime, 1% gypsum, and 1% calcium superphosphate. SD, standard deviation. T, treatment.

**Table 4:** Heavy metal contents of mushrooms cultivated on different substrates (mg kg^−1^, DM, means ± SD).

Treatment Group	As (mg kg^−1^)	Pb (mg kg^−1^)	Cd (mg kg^−1^)	Hg (mg kg^−1^)
T1	0.37 ± 0.01 c	0.30 ± 0.02 c	0.44 ± 0.03 a	0.039 ± 0.001 b
T2	0.53 ± 0.01 a	0.55 ± 0.01 a	0.46 ± 0.01 a	0.143 ± 0.006 a
T3	0.47 ± 0.01 b	0.43 ± 0.02 b	0.45 ± 0.01 a	0.140 ± 0.000 a

Notes: Different case letters above each bar represent significant differences (α = 0.05, ANOVA, LSD test). T1: 56% rice straw, 40% cow dung, 2% lime, 1% gypsum, and 1% calcium superphosphate; T2: 56% corncob, 40% cow dung, 2% lime, 1% gypsum, and 1% calcium superphosphate; T3: 56% soybean straw, 40% cow dung, 2% lime, 1% gypsum, and 1% calcium superphosphate. ANOVA, one-way analysis of variance; DM, dry matter; As, arsenic; Pb, lead; Cd, cadmium; Hg, mercury; LSD, least significant difference; SD, standard deviation; T, treatment.

## Data Availability

Data are contained within the article and Supplementary Materials.

## Supplementary Materials

The following supporting information can be downloaded at: https://www.mdpi.com/article/10.3390/life14010101/s1, Table S1: Fruiting body morphology of *Lepista sordida* mushrooms grown on different substrates. Table S2: Nutritional components of *Lepista sordida* mushrooms grown on different substrates.
