# Supplementary material for: Evaluation of Rice Straw, Corncob, and Soybean Straw as Substrates for the Cultivation of Lepista sordida
Source: Life (Basel). 2024 Jan 8;14(1):101. doi: 10.3390/life14010101 (PMC10820783; doi:10.3390/life14010101)
Supplement: Supplementary file 1 [file life-14-00101-s001.zip › life-2796565-supplementary.pdf]

Table S1. Fruiting body morphology of *Lepista sordida* mushrooms grown on different substrates (means ± SD)

Substrate	Mycelial Colonization Time (days)	Primordial Initiation Time (days)	Pileus Diameter (cm)	Pileus Thickness (cm)	Stipe Thickness (cm)	Stipe Length (cm)
T1	20.67±2.08b	16.33±0.58c	5.83±0.37b	0.97±0.09b	0.59±0.08b	5.62±0.23b
T2	21.33±0.58a	18.33±0.58b	6.02±0.39a	1.04±0.12a	0.61±0.07ab	5.89±0.54a
T3	22.33±0.58a	19.67±0.58a	6.10±0.31a	1.05±0.09a	0.63±0.06a	5.63±0.17b

Note: Different case letters represent significant differences ($p < 0.05$). T1: 56% rice straw, 40% cow dung, 2% lime, 1% gypsum, and 1% calcium superphosphate; T2: 56% corncob, 40% cow dung, 2% lime, 1% gypsum, and 1% calcium superphosphate; T3: 56% soybean straw, 40% cow dung, 2% lime, 1% gypsum, and 1% calcium superphosphate. SD, standard deviation. T, treatment.

Table S2. Nutritional components of *Lepista sordida* mushrooms grown on different substrates. (means ± SD) (g in 100 g of dry matter)

Treatment Group	Crude Fiber	Crude Fat	Crude Polysaccharide	Crude Protein	Ash
T1	8.37±0.04a	7.07±0.04a	9.13±0.01b	52.93±0.07c	13.33±0.03b
T2	6.27±0.03c	3.42±0.35b	6.03±0.01c	57.38±0.08a	12.11±0.01c
T3	8.11±0.04b	6.93±0.03a	9.39±0.01a	57.08±0.10b	13.73±0.03a

Note: Different lowercase letters above each bar in a given group indicate significant differences (α=0.05, ANOVA, LSD test). T1: treatment with 56% rice straw, 40% cow dung, 2% lime, 1% gypsum and 1% calcium superphosphate; T2: treatment with 56% corncob, 40% cow dung, 2% lime, 1% gypsum and 1% calcium superphosphate; T3: treatment with 56% soybean straw, 40% cow dung, 2% lime, 1% gypsum and 1% calcium superphosphate. ANOVA, one-way analysis of variance; LSD, least significant difference; SD, standard deviation. T, treatment.
